# Modeling Costs and Impacts of Introducing Early Infant Male Circumcision for Long-Term Sustainability of the Voluntary Medical Male Circumcision Program

**DOI:** 10.1371/journal.pone.0159167

**Published:** 2016-07-13

**Authors:** Emmanuel Njeuhmeli, Peter Stegman, Katharine Kripke, Owen Mugurungi, Gertrude Ncube, Sinokuthemba Xaba, Karin Hatzold, Alice Christensen, John Stover

**Affiliations:** 1 United States Agency for International Development, Washington, DC, United States of America; 2 Health Policy Project, Avenir Health, Glastonbury, CT, United States of America; 3 Ministry of Health and Child Care, Harare, Zimbabwe; 4 Population Services International, Harare, Zimbabwe; 5 AIDSFree Project, Jhpiego, Dar es Salaam, Tanzania; University of Ottawa, CANADA

## Abstract

Voluntary medical male circumcision (VMMC) has been shown to be an effective prevention strategy against HIV infection in males [1–3]. Since 2007, the President’s Emergency Plan for AIDS Relief (PEPFAR) has supported VMMC programs in 14 priority countries in Africa. Today several of these countries are preparing to transition their VMMC programs from a scale-up and expansion phase to a maintenance phase. As they do so, they must consider the best approaches to sustain high levels of male circumcision in the population. The two alternatives under consideration are circumcising adolescents 10–14 years old over the long term or integrating early infant male circumcision (EIMC) into maternal and child health programs. The paper presents an analysis, using the Decision Makers Program Planning Tool, Version 2.0 (DMPPT 2.0), of the estimated cost and impact of introducing EIMC into existing VMMC programs in several countries in eastern and southern Africa. Limited cost data exist for the implementation of EIMC, but preliminary studies, such as the one detailed in Mangenah, et al. [4–5], suggest that the cost of EIMC may be less than that of adolescent and adult male circumcision. If this is the case, then adding EIMC to the VMMC program will increase the number of circumcisions that need to be performed but will not increase the total cost of the program over the long term. In addition, we found that a delayed or slow start-up of EIMC would not substantially reduce the impact of adding it to the program or increase cumulative long-term costs, which should make introduction of EIMC more feasible and attractive to countries contemplating such a program innovation.

## Introduction

Voluntary medical male circumcision (VMMC) has been shown to be effective in preventing acquisition of HIV-1 infection. Three randomized controlled trial (RCT) studies in South Africa, Kenya, and Uganda demonstrated that male circumcision reduces female-to-male HIV transmission by approximately 60 percent [1–3]. In 2007, the World Health Organization (WHO) and the Joint United Nations Programme on HIV/AIDS (UNAIDS) identified 13 priority countries in eastern and southern Africa where circumcision rates were relatively low and HIV prevalence was high for scale-up of VMMC: Botswana, Kenya, Lesotho, Malawi, Mozambique, Namibia, Rwanda, South Africa, Swaziland, Uganda, Tanzania, Zambia, and Zimbabwe. The President's Emergency Plan for AIDS Relief (PEPFAR) has also extended support to Ethiopia, bringing the number of priority countries to 14 [6]. As a result, these countries have established national programs to scale up VMMC coverage to 80% among adult males (15–49 years), as part of their broader prevention agenda. Modeling conducted in 2011 projected that scaling up VMMC among males ages 15–49 to 80% coverage by 2015 and maintaining this level of coverage would avert more than 20% of new HIV infections by 2025 [7].

Significant progress has been made toward achieving the initial “catch up” targets of scaling up VMMC among sexually active adult males [8]. Thus, several countries are approaching the time when they will need to shift focus toward sustaining VMMC over the longer term, referred to as the “maintenance” or “sustainability” phase of the program. This change will require discussions about realigning program strategies and ensuring the appropriate policy environment is in place [6]. Analyses using cost and impact projections for a range of client age groups are increasingly being used to make more-informed decisions for supporting, planning, and budgeting for national VMMC programs. Once the catch-up targets have been met, sustained VMMC programs will focus on infant or adolescent circumcision or a combination of both. Policymakers will need information to help decide how the national VMMC program should be structured and how male circumcision (MC) coverage is to be maintained over the long term.

This paper uses Zimbabwe as a case study to explore the epidemiological impact and cost implications of introducing EIMC in an existing adolescent/adult VMMC program for the maintenance phase, compared with a program that sustains adult MC coverage by circumcising adolescents. This analysis should provide decision makers and VMMC program managers in priority countries with much needed information on how they might address and manage issues of sustainability.

## Methods

IRB clearance was not required for this study, because patient records were not collected or reviewed.

### DMPPT 2.0 Model

The Decision Makers Program Planning Tool, Version 2.0 (DMPPT 2.0) is described in detail in a separate manuscript in this collection [9]. Briefly, DMPPT 2.0 is a simple compartmental model implemented in Microsoft Excel 2010, designed to analyze the effects of age at circumcision on program impact and cost. DMPPT 2.0 tracks the number of circumcised males in newborns and in each five-year age group over time, taking into account age progression and mortality. The model calculates discounted VMMC program costs and HIV infections averted in the population in each year in a user-specified VMMC scale-up strategy, compared with a baseline scenario in which MC prevalence remains the same as it was. The baseline scenario assumes that traditional or other circumcisions that produced the baseline MC prevalence continue at the same rate as before the VMMC program was initiated.

### Zimbabwe Data Sources

A national DMPPT 2.0 model was created for Zimbabwe, and populated with population, mortality, and HIV incidence and prevalence projections from an external source. For the Zimbabwe country application, the authors used Spectrum/AIM (AIDS Impact Model), which projects population size, mortality, and HIV prevalence and incidence based on data empirically collected from the country. The 2013 national DMPPT 2.0 model was populated from the validated national Spectrum/AIM file obtained from the Joint United Nations Programme on HIV/AIDS (UNAIDS). Population by age and year, mortality by age and year, annual number of male births, and HIV incidence by age and year were exported from this Spectrum/AIM file into a national Zimbabwe DMPPT 2.0 file.

The Zimbabwe Ministry of Health and Child Care (MOHCC) provided the numbers of VMMCs conducted in the country each year from the beginning of the VMMC program in 2009 through December 2014, disaggregated by client age. The prevalence of male circumcision by age group in the model base year (2014) was derived from the Zimbabwe Demographic and Health Survey 2010–11 [10]. The unit cost of VMMC used in the analysis was US$122 (currency is U.S. dollars throughout), based on MOHCC data from 2014 [11]. The unit cost includes costs for personnel, outreach, commodities, training, facilities, maintenance, demand creation, waste management, transportation, quality assurance, and above-facility-level management.

### Analytical Approaches

To examine the effect of client age on the impact of scaling up VMMC, we created a series of scenarios in which MC coverage was scaled up from baseline levels to 80% for infants (zero to sixty days old) and for each five-year age group between ages 10 and 39, while all other age groups were maintained at the same level as the baseline for each scenario (see Table 1 below).

**Table 1:** Client Age-group Scenarios Used to Examine Impact of VMMC Scale-up.

Target age group	EIMC scenario, %	10–14 scenario, %	15–19 scenario, %	20–24 scenario, %	25–29 scenario, %	30–34 scenario, %	35–39 scenario, %
EIMC	80	0	0	0	0	0	0
10–14	4	80	4	4	4	4	4
15–19	5	5	80	5	5	5	5
20–24	8	8	8	80	8	8	8
25–29	11	11	11	11	80	11	11
30–34	11	11	11	11	11	80	11
35–39	11	11	11	11	11	11	80
40–44	12	12	12	12	12	12	12
45–49	12	12	12	12	12	12	12
50–54	12	12	12	12	12	12	12
55–59	12	12	12	12	12	12	12

This table shows the scenarios used to examine how the impact of VMMC scale-up changes when specific age groups receive the intervention. Scenarios are shown by column; it is not useful to read the table by row. Numbers are the target MC coverage (%) in the indicated age group to be reached by the end of 2019 and maintained thereafter. Each scenario assumed 80% MC coverage for the target age group and baseline MC coverage for all other age groups.

Each scenario scaled up MC coverage between 2015 and 2019, inclusive, by applying a linear interpolation to the baseline MC prevalence for each age group in 2014 and the target coverage by the end of 2019. After 2019, the coverage for each age group was maintained at the target level. For each age group scenario, we recorded HIV incidence in each year of the model. We then divided this annual HIV incidence by the annual HIV incidence in the reference case in which VMMC was not scaled up over baseline levels for any age group; this was called the HIV incidence ratio. Please note that the same reference case (MC prevalence maintained at baseline levels for all age groups) is used in all subsequent analyses, as well.

To compare the impact, cost, and cost-effectiveness of adolescent VMMC, EIMC, or a mixed strategy as sustainability strategies, we created three scenarios (Table 2) and projected the number of circumcisions that would need to be done between 2015 and 2051 in each (2051 is the last year of the model). In the first scenario, we assumed scaling up VMMC coverage to 80% for 10- to 34-year-olds over five years and then maintained that coverage into the future. The second scenario assumed scaling up VMMC coverage to 80% for the same age group, but also assumed scaling up infant circumcision to 80% and maintained that coverage into the future. The third scenario looked at scaling up VMMC coverage to 80% for adolescents and adults (10- to 34-year-olds), but scaling up infant circumcision only to 40% coverage, and maintained these coverage levels into the future. For each scenario, we projected the number of HIV infections averted, the number of VMMCs required, the total cost of the VMMC program, the percentage of HIV infections averted (denominator was the number of HIV infections in the baseline scenario), the number of VMMCs per HIV infection averted, and the cost per infection averted. All metrics were projected from 2015 to 2051, inclusive. Costs, numbers of VMMCs, and numbers of HIV infections averted were all discounted at a rate of 3% per year. (Different discount rates both for costs and HIV infections averted were applied in the sensitivity analysis testing different discount rates, as indicated.)

**Table 2:** Scenarios Used to Examine the Impact, Cost, and Cost-effectiveness of Three Strategies.

Target age group	10–34 scenario, %	EIMC scenario, %	Mixed scenario, %
EIMC	0	80	40
10–14	80	80	80
15–19	80	80	80
20–24	80	80	80
25–29	80	80	80
30–34	80	80	80
35–39	11	11	11
40–44	12	12	12
45–49	12	12	12
50–54	12	12	12
55–59	12	12	12

The three strategies are provision of adolescent VMMC (10–34 scenario), EIMC, or a mixed strategy during the sustainability phase. Scenarios are by column; it is not useful to read this table by row. Numbers are the target MC coverage (%) in the indicated age group to be reached by the end of 2019 and maintained thereafter. The target for the 35–39 year age group and above is the baseline MC coverage for these age groups.

If Zimbabwe decided to scale up EIMC, we wanted to look at the potential effects of introducing EIMC in the national VMMC program over different time frames, so that decision makers could assess these options for impact, cost, and cost-effectiveness. Thus we compared four scenarios that initiated EIMC and scaled it up at different rates. In the first scenario, EIMC is introduced immediately. Coverage levels for infants, adolescents, and adults are simultaneously scaled up to 80% over five years, and then maintained at that level into the future (called EIMC 5; this is the same as the EIMC scenario listed in Table 2 above). The second scenario replicates the first, but instead of EIMC scaling up to 80% coverage in five years in tandem with adolescent and adult circumcision, it is scaled up more gradually, reaching 80% over ten years (EIMC 10). In a third scenario, EIMC is introduced in 2020, after adolescent and adult circumcision scale-up to 80% coverage is achieved, and then EIMC itself is scaled up to 80% over five years (EIMC d5 –“d” stands for “delayed”). The fourth scenario in this analysis again delays introduction of EIMC until 2020, when adolescent and adult circumcision has reached 80% coverage, and then it is scaled up over ten years to reach 80% (EIMC d10).

To determine the relative cost (EIMC unit cost divided by adolescent VMMC unit cost) at which neonatal and adolescent circumcision have the same cost per lifetime HIV infection averted, we used a different analytical approach, separate from DMPPT2.0 but with the same data sources. We created a simple cohort model in which equal numbers of infants and 15-year-olds are circumcised at the same time in 2015. The total HIV infections averted for each cohort, compared to the reference scenario in which MC prevalence remains at baseline levels for each age group, is calculated as follows: for each year in each cohort’s lifetime (until and including age 85), the model adds the age-specific HIV incidence from 2015, multiplied by the effectiveness of VMMC (60%), reduced by the age-specific mortality, and discounted by 3% annually. The initial population size of the EIMC cohort is reduced by the baseline MC prevalence among adolescents, to account for the fact that when infants are circumcised through the EIMC program, those boys are preempted from being circumcised as adolescents. In other words, EIMC is replacing the baseline circumcisions that would have happened when the boys grew older, so the replacement circumcisions do not count toward the HIV infections averted. The relative unit cost at which the cost per HIV infection averted by EIMC is the same as that for adolescent VMMC is given by the cumulative discounted HIV infections averted for the EIMC cohort divided by the cumulative discounted HIV infections averted for the adolescent cohort. For a range of discount rates, investigators calculated how much lower EIMC unit costs would need to be compared to adolescent VMMC unit costs in order to have the same cost per lifetime HIV infection averted.

## Results

### Effect of Client Age on the Impact of Scaling Up VMMC

**Fig 1:**
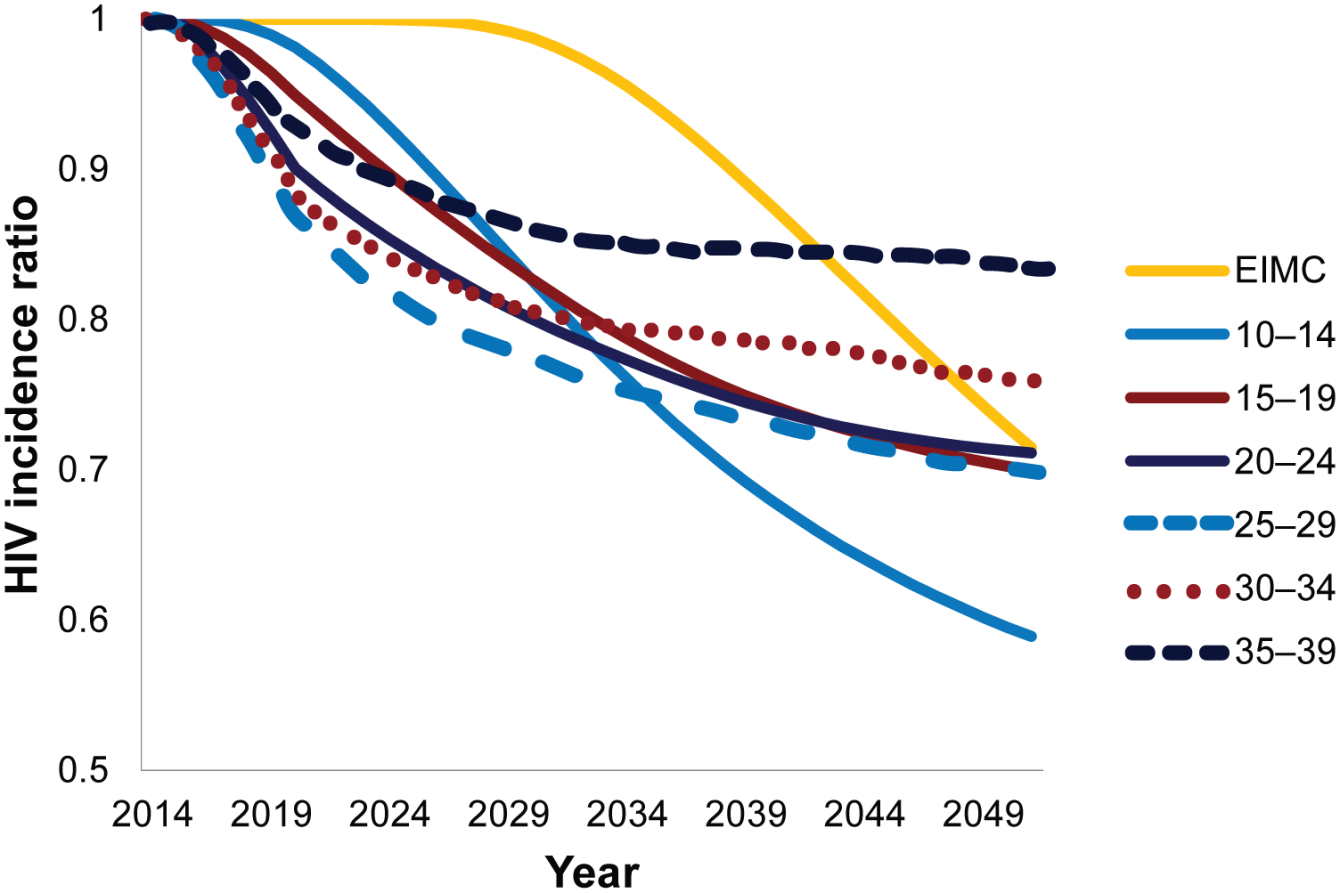
Modeled relative reduction in HIV incidence with provision of VMMC by age group, 2015–2051.

Fig 1 presents the impact of providing VMMC to different age groups of clients on the incidence of HIV in Zimbabwe over time, in comparison with the HIV incidence that results from a scenario in which VMMC is maintained at baseline levels. Each line represents a fictitious scenario in which VMMC was provided only to the indicated age group and the HIV incidence among the entire population was tracked over time. With each successively younger age group, the overall impact on HIV incidence is projected to be greater. Also, with the age groups older than 15 years, which are already sexually active, the impact is greater in the early years as the program scales up to 80% coverage, but then it begins to level off. The impact of VMMC among 10- to 14-year-olds and infants is delayed until these boys become sexually active, and then it increases steeply. If the analysis were to be projected beyond 2051, the yellow EIMC line would cross the line that represents circumcising 10- to 14-year-olds, suggesting a slightly greater impact over time.

### Circumcision of Infants, Adolescents, or Both as Strategies for Maintaining MC Prevalence over the Long Term

**Fig 2:**
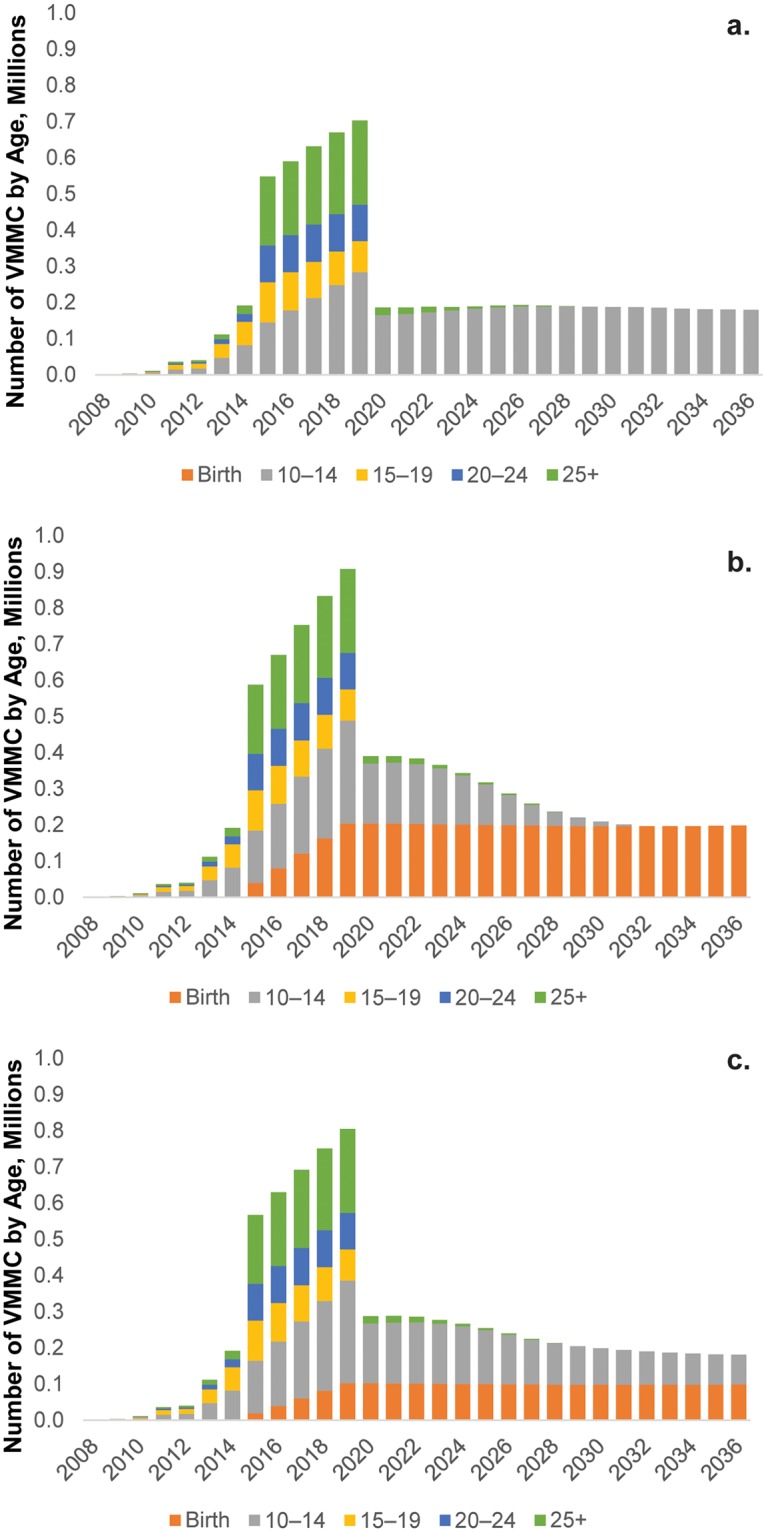
Annual number of VMMCs to be conducted under three scenarios. These scenarios are VMMC scaled up to (a) 80% coverage among 10- to 34-year-olds, (b) 80% coverage among 10- to 34-year-olds + 80% EIMC, (c) 80% coverage among 10- to 34-year-olds + 40% EIMC.

Fig 2a, 2b and 2c present three scenarios of VMMC scale-up in Zimbabwe. In Fig 2a, EIMC is not introduced and there is an intensive scale-up of VMMC among adolescents and adults (10–34 years old) to 80% coverage in five years, reaching a peak annual volume in 2019 of nearly 600,000 circumcisions. After 80% coverage has been achieved, the number of circumcisions among age groups 15 years and older drops off, as saturation is achieved in these groups; the maintenance phase of the program involves circumcising those individuals in the male population who move into the 10–14 year age group every year. On average, this translates into just under 190,000 circumcisions annually to maintain coverage levels.

In Fig 2b, EIMC is introduced in the VMMC program and scaled up to 80% coverage in five years simultaneously with adolescents and adults. Adding infants gradually increases the total number of males requiring circumcision services annually, growing to a 29% increase—nearly 205,000 infants—by 2019. This translates into an overall circumcision target of more than 900,000 males in that year. As with the previous scenario, once 80% coverage is achieved, circumcisions among those ages 15 and older are no longer needed. Initially, maintaining coverage levels involves circumcising infants and 10- to 14-year-olds. However, because of the introduction of EIMC, after about 10 to 12 years, the program will have achieved saturation among 10- to 14-year-olds and the long-term maintenance of the program will focus on circumcising infants. Although adding EIMC increases the overall number of circumcisions that need to be performed from 2015 to 2051 by 22%, the impact—in terms of the number of infections averted over this same period—is increased by only 1% (Table 3).

**Table 3:** Impact of Adding EIMC on HIV Infection Rates.

Zimbabwe	10–34	10–34+ EIMC	% difference
Infections averted	266,000	268,000	1
Number of MCs	9,000,000	11,000,000	22
% Infections averted	50	50	0
VMMC per infection averted	23	29	24

A mixed strategy is presented in Fig 2c, where EIMC is scaled up to just 40% coverage while adults and adolescents achieve 80% coverage over five years. This option may be adopted by some countries. A more modest provision of services to infants does not produce as quick an expansion in the numbers of males circumcised every year. By 2019, the number is up to more than 102,000 infants, or a 14.5% increase in the overall number of circumcisions performed. This represents a total of just over 805,000 circumcisions in 2019. After the five-year scale-up period, the same pattern emerges, in which circumcisions for males ages 15 years and older cease, and the program is maintained by circumcising infants and 10- to 14-year-olds.

### Cost and Cost-effectiveness of EIMC versus Adolescent VMMC as Sustainability Strategies

The costs and cost drivers of adolescent and adult VMMC are well understood, but currently there is little empirical data on the unit costs of EIMC in sub-Saharan Africa, and there is a lack of any data directly comparing the cost of EIMC with adolescent and adult VMMC. Only when EIMC is scaled up broadly will a better understanding of costs be possible. Therefore, the investigators conducted a sensitivity analysis with different assumptions of the relative EIMC cost compared with the cost of adolescent/adult VMMC. Table 4 shows the cost and cost-effectiveness of introducing EIMC in the existing VMMC program in Zimbabwe, using different ratios of EIMC unit cost compared with adolescent/adult VMMC unit cost. We based these calculations on the number of VMMCs and infections averted for the 10–34 and 10–34 + EIMC scenarios from Table 3, above. The results presented in Table 4 show that adding EIMC can increase the total cost of the program and cost per HIV infection averted if the cost of EIMC is the same as or 80% of the cost of adolescent and adult VMMC. When the EIMC cost is 50% or 25% of the adolescent/adult VMMC cost, adding EIMC to the program lowers the program’s total cost as well as the cost per HIV infection averted. At 25% of the adult/adolescent cost, adding EIMC leads to substantial (24%) cost savings. Given the likelihood that EIMC will be integrated in existing maternal and child health programs where services are delivered by nurses and midwives, and in light of costing data from Mangenah, et al. [4], we anticipate that the cost of EIMC will be about half the cost of adolescent/adult VMMC [4]. The remaining analyses in this paper assume this 50% lower cost of EIMC compared with that of adolescent VMMC.

**Table 4:** Cost and Cost-effectiveness of Adding EIMC.

Cost of EIMC/Cost of Adolescent VMMC (%)	Model output	10–34	10–34+ EIMC	% difference
100%	Total cost	$747,000,000	$957,000,000	25
	Cost per HIV infection averted	$4,127	$5,256	24
80%	Total cost	$747,000,000	$858,000,000	14
	Cost per HIV infection averted	$4,127	$4,713	13
50%	Total cost	$747,000,000	$710,000,000	–5
	Cost per HIV infection averted	$4,127	$3,898	–6
25%	Total cost	$747,000,000	$586,000,000	–24
	Cost per HIV infection averted	$4,127	$3,219	–25

### Timing of Introduction and Scale-up of EIMC

**Fig 3:**
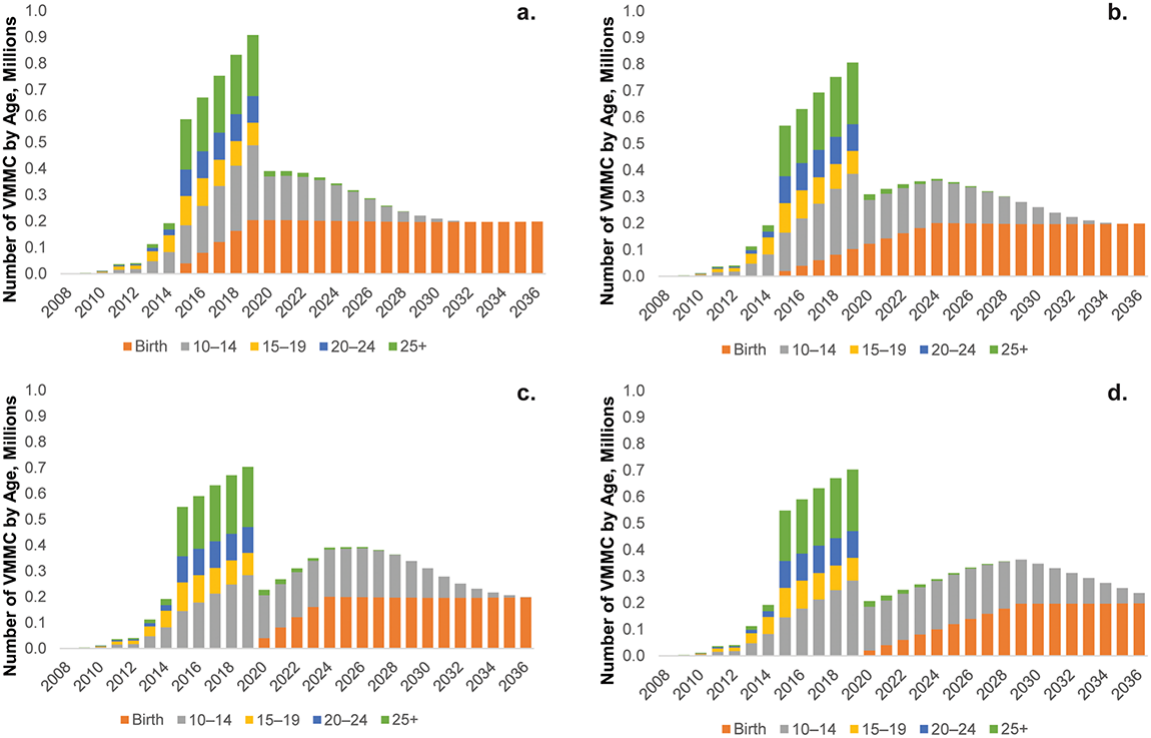
Annual number of VMMCs to be conducted under four timing scenarios. (a) EIMC introduced immediately and scaled up to 80% over 5 years (EIMC 5), (b) EIMC introduced immediately and scaled up to 80% over 10 years (EIMC 10), (c) EIMC introduced after 5 years and scaled up to 80% over 5 years (EIMC d5), (d) EIMC introduced after 5 years and scaled up to 80% over 10 years (EIMC d10).

The previous scenarios modeled what VMMC scale-up and maintenance would look like if adolescent, adult, and early infant male circumcision were scaled up at the same time. Some countries, however, are considering either delaying the start of EIMC or taking a longer time to scale up. Fig 3a repeats the scenario above where EIMC is introduced immediately and the scale-up of adult, adolescent, and EIMC would take place over a five-year period (EIMC 5). In Fig 3b, EIMC is introduced immediately but is scaled up more gradually over a 10-year period (EIMC 10). Fig 3c and 3d present the pattern and number of total circumcisions required if the introduction of EIMC in an existing VMMC program is delayed until after 80% coverage has been attained. In Fig 3c, the introduction of EIMC is delayed for five years, and then it is scaled up to 80% coverage over a five-year period(EIMC d5). In Fig 3d, the introduction of EIMC is delayed for five years until after the scale-up of adolescent and adult VMMC to 80% coverage, and then it is scaled up more gradually, over a period of 10 years (EIMC d10).

Table 5 presents the cost and cost-effectiveness data from each of the study’s possible EIMC introduction and scale-up scenarios for Zimbabwe: EIMC 5, EIMC 10, EIMC d5, and EIMC d10. The model reveals that there is very little difference between each of the introductory and scale-up scenarios. Generally, each averts the same number of infections and would require approximately the same number of circumcisions to be performed over the period. There is a marginal (1%–4%) increase in the total cost for strategies in which introduction of EIMC is delayed as opposed to being started immediately. There is a slightly lower (3%) number of VMMCs per infection averted when the introduction of EIMC is delayed, and there is only a marginal increase of $115 if the cost per infection averted increases from EIMC 5 to EIMC d10. Thus, either extending or delaying scale-up of EIMC does not substantially reduce the impact or increase the costs.

**Table 5:** Cost and Cost-effectiveness of EIMC Start-up, by Scenario.

Zimbabwe	EIMC5	EIMC10	EIMCd5	EIMCd10
Infections averted	268,000	267,000	267,000	267,000
# MCs	11,000,000	11,000,000	11,000,000	11,000,000
Total cost*	$710,000,000	$719,000,000	$731,000,000	$738,000,000
% Infections averted	50	50	50	50
VMMC per infection averted	29	29	28	28
Cost per infection averted	$2,649	$2,693	$2,738	$2,764

*Based on the assumption that the cost of EIMC is 50% of the cost of adolescent and adult VMMC

### At What Unit Cost Is the Cost per HIV Infection Averted the Same for EIMC as It Is for Adolescent VMMC?

Table 6 presents the cost ratio at which the lifetime cost-effectiveness is equal for EIMC and adolescent VMMC across different countries and using different discount rates. The percentages shown in the table are the unit costs of EIMC as a percentage of the unit cost of adolescent VMMC. In Zimbabwe, for example, if the discount rate is 3% and the costs of $122 per MC for adolescents and adults and $69.20 (57% of $122) for EIMC are considered, then the lifetime cost-effectiveness of the two circumcision strategies will be equal. For countries with lower baseline MC prevalence, such as Zimbabwe, the cost at which EIMC is equally cost-effective as adolescent VMMC is relatively high. Conversely, for countries such as Tanzania and South Africa with much higher MC prevalence as a result of traditional MC, the cost of EIMC must be lower for its lifetime cost-effectiveness to be equal to that of adolescent VMMC. Thus, the cost point at which there is equilibrium between the lifetime cost-effectiveness of EIMC and adolescent VMMC is dependent upon the discount rate used and the country’s baseline MC prevalence among adolescents.

**Table 6:** Cost Ratios for EIMC and VMMC by Country.

Country	Baseline MC (15–19-year-olds) (%)	Discount rate (%)				
		0%	3%	5%	7%	10%
Tanzania	40	52	33	25	19	12
South Africa	31	65	42	31	23	15
Uganda	22	69	44	33	25	16
Malawi	11	82	53	39	30	20
Zimbabwe	5	88	57	43	32	21
Swaziland	4	86	55	41	31	21

## Discussion

This paper provides important modeling of the potential cost and impact of introducing EIMC in existing national VMMC programs. The research was conducted using data from applications of the DMPPT 2.0 model in multiple countries, but focused specifically on Zimbabwe as a case study. The modeling explores scenarios countries may pursue for the long-term maintenance of the VMMC program: MC coverage may be maintained in the long term by circumcising adolescents or infants or a combination of the two. These scenarios affect the overall cost and cost-effectiveness of the program, but they do not substantially affect the impact on new HIV infections, because both infants and adolescents are circumcised before reaching sexual maturity. The cost and cost-effectiveness of introducing EIMC in a VMMC program are functions of the relative cost of EIMC compared to that of VMMC, the baseline levels of circumcision among adolescents, and the discount rate applied.

The study has some limitations. The general limitations of the DMPPT 2.0 model are discussed in the methods paper in this collection [9] and also apply to this analysis. The unit cost of EIMC compared to adolescent and adult VMMC is not known. Despite this uncertainty, for the current analysis, it is assumed that EIMC may cost 50% or less of adolescent and adult VMMC. EIMC is a simpler procedure than adolescent and adult VMMC. It requires no suturing, has fewer complications [12], and will be an integrated service provided by existing personnel not dedicated to EIMC alone, whereas adult/adolescent VMMC is often implemented as a vertical program. However, the fact is that the unit cost of EIMC, compared to that of adolescent and adult VMMC, will likely vary by country. The numbers reported for total cost should be considered indicative for comparison purposes, but are not intended to provide budget projections, because the actual costs of EIMC in different settings are unknown. Likewise the numbers of HIV infections averted and the costs per HIV infection averted are dependent on future HIV incidence projections. Just as actual future HIV incidence is unknown and is more uncertain the further into the future it is projected, so these numbers should be considered for comparison purposes only.

This analysis has important implications for sustainability of VMMC programs once the initial coverage target has been met. The findings presented here suggest that consideration should be given to the cost of EIMC compared to that of adolescent and adult MC. The modeling data show that introducing EIMC in an existing VMMC program, while increasing the overall number of MCs required, does not necessarily increase overall cost. If the unit cost of EIMC is 50% or less of the cost of adolescent and adult VMMC, there is actually a cost savings and the cost per HIV infection averted is lower. However, as the cost of EIMC increases relative to that of adolescent and adult VMMC, the cost savings erodes, and the introduction of EIMC can actually increase the overall cost and decrease the cost-effectiveness of the program. If the unit cost were equal, the total cost of EIMC as well as the cost per infection averted would be about 20%–25% more than a program consisting entirely of adolescent and adult VMMC.

This study’s modeling of the introduction of EIMC in existing national VMMC programs indicated that the timing (whether immediate or delayed) and the intensity of scale-up (over five or ten years) had only a marginal impact on the number of infections averted, on the cumulative cost of the program, and on the number of VMMCs required to avert a single HIV infection. This is good news for countries that are delaying EIMC because of the serious challenge its introduction might pose while they are at the same time trying to reach coverage targets for adults and adolescents.

Furthermore, for countries with lower baseline MC prevalence, such as Zimbabwe, the cost at which EIMC is equally as cost-effective as adolescent VMMC is relatively high. Conversely, for countries such as Tanzania and South Africa, where MC prevalence is much higher, the cost of EIMC must be lower for its lifetime cost-effectiveness to be equal to that of adolescent VMMC. Thus, the cost point at which there is equilibrium between the lifetime cost-effectiveness of EIMC and adolescent VMMC is dependent upon the discount rate used and the country’s baseline MC prevalence among adolescents.

However, the cost savings and impact of expanding EIMC is likely underestimated, because our analysis does not account for the full extent of the benefits of infant male circumcision, such as the prevention or reduction of urinary tract infections (a risk factor for neonatal sepsis), sexually transmitted infections, phimosis, paraphimosis, and other medical conditions that could be prevented through MC at an earlier life stage. Integrating the provision of EIMC within existing maternal and child health services in resource-limited settings is likely to be easier and more acceptable to the government and ministry of health than maintaining parallel, vertical adult and adolescent VMMC programs, because EIMC can be combined with existing routine postnatal or vaccination services. Additionally, a vertical model requires dedicated staff redirected from the health delivery system to conduct MCs, whereas EIMC at the primary healthcare level could be delivered by current personnel who could continue to provide maternal and child health services alongside EIMC.

Zimbabwe is currently completely dependent on external funding for its vertical adult and adolescent VMMC program. If external funding ceases, the Government will likely not be able to sustain a vertical adolescent VMMC program at the current costs, even if the number of adolescents aging into the 10–14 year age group to be circumcised is much lower than the number currently being circumcised in the scale-up phase. The government health providers will then have to look for less costly, more fully integrated models to sustain VMMC efforts, while improving existing health systems for maternal and child health.

In addition, the model does not account for broader social, cultural, and logistical barriers to EIMC acceptability and implementation. However, acceptance of EIMC will likely rise as greater numbers of procedures are performed and adult and adolescent VMMC continues to be scaled up. With increased uptake, the unit costs of EIMC are likely to decrease further. Demand creation efforts and costs are important to consider to ensure efficient use of EIMC services and achievement of lower unit costs, owing to economies of scale. When selecting a strategy to sustain national VMMC programs, countries will need to consider the modeling and analysis in this paper in the context of other factors that may influence the course of decision making.

In conclusion, the analyses presented here provide some factors for countries to consider when determining whether to base their long-term VMMC programs on EIMC, adolescent VMMC, or some combination of the two. Differences in epidemiological impact in terms of HIV infections averted are negligible between the different strategies. The relative total cost and cost-effectiveness of the different strategies are largely dependent on the relative cost of EIMC compared with that of adolescent VMMC, so more research is needed in terms of collecting comparable costs for sustainable models of EIMC and adolescent VMMC implementation. Cost and cost-effectiveness, while important, must be considered alongside other factors, such as acceptability of either approach to parents and health providers, health systems impacts of different implementation models, and feasibility concerns.
