# Correction: Modeling Costs and Impacts of Introducing Early Infant Male Circumcision for Long-Term Sustainability of the Voluntary Medical Male Circumcision Program

**DOI:** 10.1371/journal.pone.0169298

**Published:** 2016-12-22

**Authors:** Emmanuel Njeuhmeli, Peter Stegman, Katharine Kripke, Owen Mugurungi, Gertrude Ncube, Sinokuthemba Xaba, Karin Hatzold, Alice Christensen, John Stover

There is an error in the Funding statement. The number for the Cooperative Agreement Project SOAR (Supporting Operational AIDS Research) is incorrect. The correct number is OAA-A-14-00060.
